# Investigating the causal relationship between inflammation and multiple types of hearing loss: a multi-omics approach combining Mendelian randomization and molecular docking

**DOI:** 10.3389/fneur.2024.1422241

**Published:** 2024-11-28

**Authors:** Jingqi Zhang, Tao Guo, Yaxin Chen, Xiangjin Wang, Lijiao Wu, Hui Xie

**Affiliations:** ^1^Hospital of Chengdu University of Traditional Chinese Medicine, Chengdu, China; ^2^School of Sports Medicine and Health, Chengdu Sport University, Chengdu, China

**Keywords:** sensorineural hearing loss, sudden idiopathic hearing loss, Mendelian randomization, inflammation, multi-omics

## Abstract

**Background:**

Hearing loss affects over 10% of the global population. Inflammation is a key factor in hearing loss caused by noise, infection, and aging, damaging various hearing-related tissues (e.g., spiral ligament, stria vascularis). Mendelian randomization (MR) can help identify potential causal relationships and therapeutic targets.

**Methods:**

We conducted MR analyses on 91 inflammatory proteins (*n* = 14,824) and genome-wide association study results for various hearing loss types in European ancestry populations, including sensorineural hearing loss (SNHL; *n*cases = 15,952, *n*controls = 196,592), sudden idiopathic hearing loss (SIHL; *n*cases = 1,491, *n*controls = 196,592), and other hearing loss (OHL; *n*cases = 4,157, *n*controls = 196,592). Additionally, hearing loss with difficulty in hearing (*n*cases = 14,654, *n*controls = 474,839) served as a validation set. To predict inflammatory protein-enriched pathways and tissues, we performed enrichment analysis, functional annotation, and tissue analyses using “OmicsNet2.0” and “FUMA” platforms. We also combined “CoreMine” and molecular docking to explore potential drugs targeting inflammatory proteins and investigate binding efficacy.

**Results:**

CCL19 was identified as a common risk factor for SNHL and OHL, which was validated in the hearing loss with difficulty in hearing dataset. Tissue analysis revealed that SIHL-related inflammatory proteins were enriched in the amygdala. Multi-omics research indicated associations between inflammatory proteins and neurodegenerative diseases. Molecular docking studies suggested that Chuanxiong Rhizoma and Uncariae Ramulus Cumuncis are potential drugs for targeting CCL19.

**Conclusion:**

This study identified CCL19 as a common risk factor for various types of hearing loss through MR analysis, highlighting the crucial role of inflammatory proteins in hearing loss. The enrichment of related inflammatory proteins in the amygdala and their association with neurodegenerative diseases provide new insights into the mechanisms of hearing loss.

## 1 Introduction

According to 2015 statistics, hearing loss affects over 10% of the world’s population, making it the second most common disability after anemia (1). The increasing noise pollution and the use of ototoxic drugs (aminoglycoside antibiotics, loop diuretics, anti-tumor drugs, etc.) (2) have also exacerbated hearing loss, not only affecting health and quality of life but also causing a significant economic burden.

Multiple factors can lead to hearing loss, including genetic factors (3), noise pollution (4), infections (5), aging (6), and ototoxic drugs (7, 8). Inflammation plays a crucial role in these processes. For instance, the expression of inflammatory factor IL-6 has been observed in the spiral ligament, stria vascularis, and spiral ganglion neurons following noise exposure (9). Infection-induced inflammatory states can damage inner ear hair cells and spiral ganglion neurons (10). Age-related hearing loss (ARHL) is associated with low-grade inflammation (11), while ototoxic drugs like cisplatin increase reactive oxygen species (ROS) levels and inflammatory factor expression, leading to hearing loss (12). Existing studies have explored the relationship between certain inflammatory factors and hearing loss (13), but have not clearly established causal links and are limited by factors such as low protein coverage, small cohort sizes, and potential confounding biases.

Recent advances in genomics have provided powerful tools for investigating the genetic basis of complex diseases like hearing loss. Mendelian randomization (MR) uses genetic variants as instrumental variables to infer causal relationships between exposures and outcomes (14), helping to overcome limitations of observational studies by reducing confounding and reverse causation (15). Previous studies have employed MR to explore the causal relationships between white blood cell count and sudden sensorineural hearing loss (16), serum lipids and ARHL loss (17), and mitochondrial proteins and inflammatory diet with sensorineural hearing loss (SNHL) (18, 19).

Our study utilized MR to investigate the relationship between 91 inflammatory proteins and various types of hearing loss. Additionally, multi-omics analysis and tissue analysis were integrated to explore the mechanisms by which inflammatory proteins cause hearing loss and identify enriched tissues. Finally, potential drug discovery and molecular docking were combined to identify novel drugs targeting inflammatory proteins for the treatment of hearing loss.

## 2 Methods

**Figure 1:**
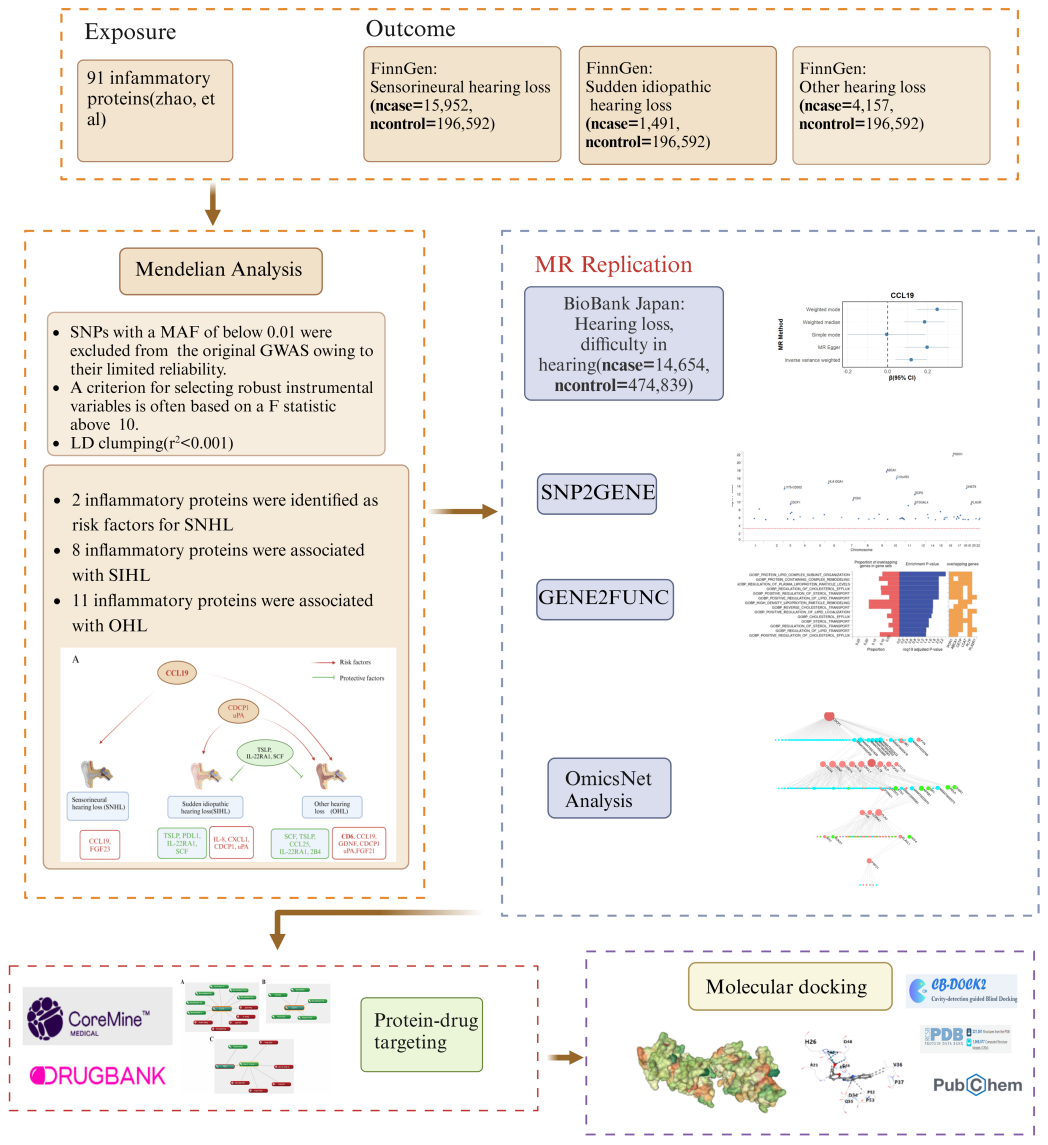
Research flow diagram. MR, Mendelian randomization; SNHL, sensorineural hearing loss; SIHL, sudden idiopathic hearing loss; OHL, other hearing loss.

The study followed the STROBE-MR checklist for standardized MR analysis (20) (Supplementary Table S1). The overall study design is presented in Figure 1. The exposure data was derived from 91 inflammatory proteins (14,824 participants) provided by Zhao et al. (21). The outcome data for three types of hearing loss were obtained from the FinnGen database.^1^ SNHL (*n*case = 15,952, *n*control = 196,592) was defined as hearing loss due to the inner ear or sensory organ (cochlea and associated structures) or vestibulocochlear nerve, and patients were diagnosed using ICD-10 codes H90.3, H90.4, and H90.5 (22). Sudden idiopathic hearing loss (SIHL) (*n*case = 1,491, *n*control = 196,592) was diagnosed using ICD-10 code H91.2. Other hearing loss (OHL) (*n*case = 4,157, *n*control = 196,592) includes conditions such as deaf mutism, other specified/unspecified hearing loss, ototoxic hearing loss, presbycusis, etc. All data pertain to European populations. The validation data was sourced from the Biobank Japan database for hearing loss, difficulty in hearing (*n*case = 14,654, *n*control = 474,839) (23). Detailed information of the data can be found in Supplementary Table S2.

Subsequently, we utilized the functional mapping and annotation of genome-wide association studies (FUMA) platform, specifically its “SNP2GENE” and “GENE2FUNC” tools, to perform functional annotation, gene-based association, and tissue analyses on SIHL-related proteins. Through multi-omics analysis, we identified miRNAs and transcription factors involved in the crosstalk between inflammatory proteins, as well as explored potential signaling pathways. To discover effective therapeutic agents, we employed “CoreMine” to mine for drugs targeting key proteins. Additionally, we used molecular docking to evaluate the binding capacity of small molecule drugs to these critical proteins.

### 2.1 Mendelian randomization analysis

This study followed the core assumptions of MR analysis (Supplementary Figure S1). The following criteria were used to select genetic variants associated with inflammatory proteins: (i) we selected genetic variants strongly associated with inflammatory proteins (*p* < 5 × 10^−6^) as instrumental variables and excluded any variants that were directly associated with SNHL, SIHL, OHL, or difficulty in hearing in genome-wide association studies; (ii) to ensure independence between single-nucleotide polymorphisms (SNPs), linkage disequilibrium (LD) clustering was performed, with a 10,000 kb window and *r*^2^ < 0.001 as the filtering standard (24); (iii) SNPs with a minor allele frequency (MAF) below 0.01 were excluded due to their limited reliability (25); and (iv) a criterion for selecting robust instrumental variables is often based on an *F*-statistic above 10, calculated as: *R*^2^ = 2 × EAF × (1 − EAF) × beta^2^; *F* = *R*^2^ × (*N* − 2)/(1 − *R*^2^) (26).

The “TwoSampleMR” software package was used to perform MR analysis. Multiple methods were used to evaluate the causal effects of genetic variants on hearing loss, including simple mode, weighted mode, weighted median, and MR-Egger, as well as inverse variance weighted (IVW) method, which is considered the primary method (27). The *Q* statistic was used to evaluate the heterogeneity of genetic instruments (28). The MR-Egger intercept was used to test for directional pleiotropy, and results with evidence of pleiotropy were excluded. False discovery rate (FDR) correction was used to reduce the likelihood of false positive discoveries, and results with FDR <0.05 were considered significant. All analyses were performed in R software (version 4.2.3).

### 2.2 Functional annotation, gene-based association and tissue analyses

We utilized FUMA platform to prioritize the most likely causal SNPs and genes, and to provide gene-based, pathway, and tissue enrichment results (29). FUMA integrates information from 18 biological data repositories and tools. We employed FUMA’s “SNP2GENE” tool to further analyze previous results and screen for priority genes. The “GENE2FUN” tool was used for functional enrichment and disease pathway analyses of the identified genes. Additionally, we conducted tissue expression analysis using MAGMA within “SNP2GENE.” MAGMA tests the relationship between tissue-specific gene expression and disease gene associations through gene property analysis.

The “SNP2GENE” analysis parameters were set as follows: the maximum *p*-value threshold for lead SNPs was 5 × 10^−8^, with a maximum *p*-value cutoff of 0.05. To define independent significant SNPs, the *r*^2^ threshold was set to 0.6; a second *r*^2^ threshold of 0.1 was used to define lead SNPs (29–31). The 1,000 genomes phase 3 European population was selected as the reference panel, matching the study population’s background. We opted to include non-GWAS tagged SNPs from the reference panel for LD analysis, enhancing the precision of LD assessment. The MAF was set to 0, allowing consideration of variants at all frequencies, and the maximum distance between LD blocks to be merged into a locus was set to 250 kb (32). These parameter settings allowed for a comprehensive and stringent functional annotation and mapping of the GWAS results. The maximum *p*-value threshold ensured focus on the most significant association signals, while the *r*^2^ thresholds helped identify independent significant SNPs and lead SNPs. The 250 kb LD block merging distance aided in defining potentially functionally related genomic regions.

### 2.3 Multi-omics network analysis

Multi-omics analysis was performed using “OmicsNet2.0”^2^ (33). The input data consisted of 17 proteins associated with SNHL, SIHL, and OHL, as identified through MR analysis. The corresponding Gene IDs for these proteins were obtained from the National Center for Biotechnology Information (NCBI).^3^

The “protein–protein” interactions were established based on the “InnateDB” database, while the “transcription factor-target gene” relationships were determined using the “TRRUST” database. The “miRNA-mRNA” interactions were derived from the “miRTarBase” database (34). By integrating these diverse data sources, a comprehensive multi-omics network was constructed, with the resulting visualization output in the form of a tree diagram.

This multi-omics approach allowed for the exploration of the complex relationships between proteins, transcription factors, miRNAs, and their target genes, providing a systems-level understanding of the molecular mechanisms underlying hearing loss. The integration of multiple omics data types enabled the identification of key regulatory pathways and potential therapeutic targets, offering valuable insights into the pathogenesis of SNHL, SIHL, and OHL.

### 2.4 Drug repurposing opportunities

To explore the potential of the core protein identified through multiple screening steps as a drug target and to investigate drug repurposing opportunities, the “CoreMine” database^4^ (35) was utilized. “CoreMine” was searched for potential drug targets (including both Western and Chinese medicines) related to the core protein, aiming to assess the feasibility of targeting this protein for therapeutic purposes.

The drugs identified through the “CoreMine” search were further validated using the “DrugBank” database^5^ to ensure their accuracy and to obtain information on their mechanisms of action and disease associations. This validation step helped to confirm the relevance of the identified drugs to the core protein and their potential for repurposing in the context of hearing loss treatment.

By leveraging the information from both “CoreMine” and “DrugBank,” a comprehensive understanding of the drug targeting potential of the core protein was obtained. This approach not only considered the direct targeting of the core protein but also explored the targeting of its associated pathways and regulatory mechanisms, as identified through the multi-omics network analysis.

### 2.5 Molecular docking analysis

Following the identification of traditional Chinese medicines (TCMs) targeting the inflammatory proteins through “CoreMine,” molecular docking simulations were conducted between the main active components of these TCMs and inflammatory proteins. The effective components of TCMs were retrieved from the TCMSP database, selecting those that met the criteria of oral bioavailability (OB ≥30%) and drug-likeness (DL ≥0.18) (36).

The small molecule structures of the qualifying TCM components were obtained from the PubChem database.^6^ The UniProt ID for inflammatory proteins was acquired from the UniProt database,^7^ and its structural data was downloaded from the RCSB Protein Data Bank (PDB, https://www.rcsb.org/).

Prior to molecular docking, energy minimization was performed on the small molecules using Chem3D software (version 22.0) to achieve more stable conformations. The protein structure underwent preprocessing in CB-Dock, which included removing water molecules, adding hydrogen atoms, and repairing missing residues. Molecular docking simulations were carried out using the CB-Dock2 online tool^8^ (37).

This comprehensive approach allowed for a systematic evaluation of potential interactions between TCM components and inflammatory proteins, providing insights into possible therapeutic mechanisms and drug repurposing opportunities for hearing loss treatment.

## 3 Results

### 3.1 Mendelian randomization analysis results

**Figure 2:**
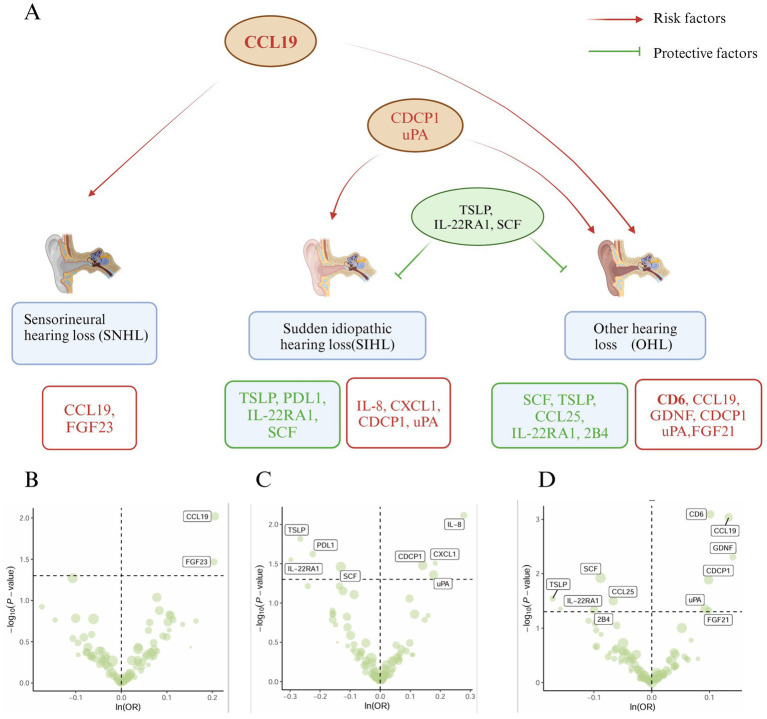
Mendelian analysis results of inflammatory proteins and hearing loss (SNHL, SIHL, OHL). **(A)** Relationship plot of inflammatory proteins and hearing loss (SNHL, SIHL, OHL). **(B)** Volcano plot of risk proteins (CCL19, FGF23) associated with SNHL. **(C)** Volcano plot of protective proteins (TSLP, PDL1, IL-22RA1, SCF) and risk proteins (IL-8, CXCL1, CDCP1, uPA) associated with SIHL. **(D)** Volcano plot of protective proteins (SCF, TSLP, CCL25, IL-22RA1, 2B4) and risk proteins (CD6, CCL19, GDNF, CDCP1, uPA, FGF21) associated with OHL.

In the MR analysis, the IVW method was used as the standard to identify 17 inflammatory proteins associated with hearing loss. Among them, SNHL and OHL shared a common risk factor (CCL19), while SIHL and OHL had common risk factors (CDCP1, uPA) and common protective factors (TSLP, IL-22RA1, SCF) (Figure 2A). Two inflammatory proteins were identified to be associated with SNHL, both of which were risk factors for SNHL: CCL19 (*β* = 0.21, 95% CI = 0.05–0.36, *p* = 0.01), FGF23 (*β* = 0.20, 95% CI = 0.02–0.39, *p* = 0.03) (Figure 2B) (Supplementary Table S3A). Eight inflammatory proteins were found to be related to SIHL, with four being protective factors for SIHL: TSLP (*β* = −0.27, 95% CI = −0.48 to −0.05, *p* = 0.015), PDL1 (*β* = −0.22, 95% CI = −0.42 to −0.03, *p* = 0.024), IL-22RA1 (*β* = −0.30, 95% CI = −0.56 to −0.03, *p* = 0.028), SCF (*β* = −0.13, 95% CI = −0.25 to −0.01, *p* = 0.035). The other four were risk factors for SIHL: IL-8 (*β* = 0.28, 95% CI = 0.07–0.48, *p* = 0.008), CXCL1 (*β* = 0.18, 95% CI = 0.02–0.35, *p* = 0.031), CDCP1 (*β* = 0.14, 95% CI = 0.01–0.27, *p* = 0.033), uPA (*β* = 0.18, 95% CI = 0.01–0.35, *p* = 0.043) (Figure 2C) (Supplementary Table S4A).

Eleven inflammatory proteins were associated with OHL, with five being protective factors: SCF (*β* = −0.09, 95% CI = −0.16 to −0.01, *p* = 0.01), TSLP (*β* = −0.17, 95% CI = −0.33 to −0.02, *p* = 0.029), CCL25 (*β* = −0.07, 95% CI = −0.13 to −0.01, *p* = 0.031), IL-22RA1 (*β* = −0.16, 95% CI = −0.31 to −0.004, *p* = 0.044), 2B4 (*β* = −0.10, 95% CI = −0.20 to −0.002, *p* = 0.046). Six proteins were identified as risk factors for OHL: CD6 (*β* = 0.10, 95% CI = 0.04–0.16, *p* = 0.0008, *p*_FDR_ = 0.04), CCL19 (*β* = 0.13, 95% CI = 0.05–0.21, *p* = 0.0009, *p*_FDR_ = 0.04), GDNF (*β* = 0.14, 95% CI = 0.04–0.24, *p* = 0.005), CDCP1 (*β* = 0.10, 95% CI = 0.02–0.18, *p* = 0.01), uPA (*β* = 0.09, 95% CI = 0.002–0.18, *p* = 0.044), FGF21 (*β* = 0.10, 95% CI = 0.001–0.196, *p* = 0.047) (Figure 2D). Among these, CD6 and CCL19 exhibited higher significance (*p*_FDR_ < 0.05) (Supplementary Table S5A). The *F*-statistics for the MR studies of SNHL, SIHL, and OHL were greater than 10 (Supplementary Tables S3B–S5B). The simple mode, weighted mode, weighted median, and MR-Egger methods generally supported the results of the IVW method (Supplementary Figures S2–S4), and the *p*-value of the intercept term in MR-Egger regression >0.05, no horizontal pleiotropy was observed (Supplementary Tables S3C–S5C). The heterogeneity analysis is provided in Supplementary Tables S3D–S5D, and the leave-one-out analysis results (Supplementary Figures S5, S6) indicated the robustness of our study.

**Figure 3:**
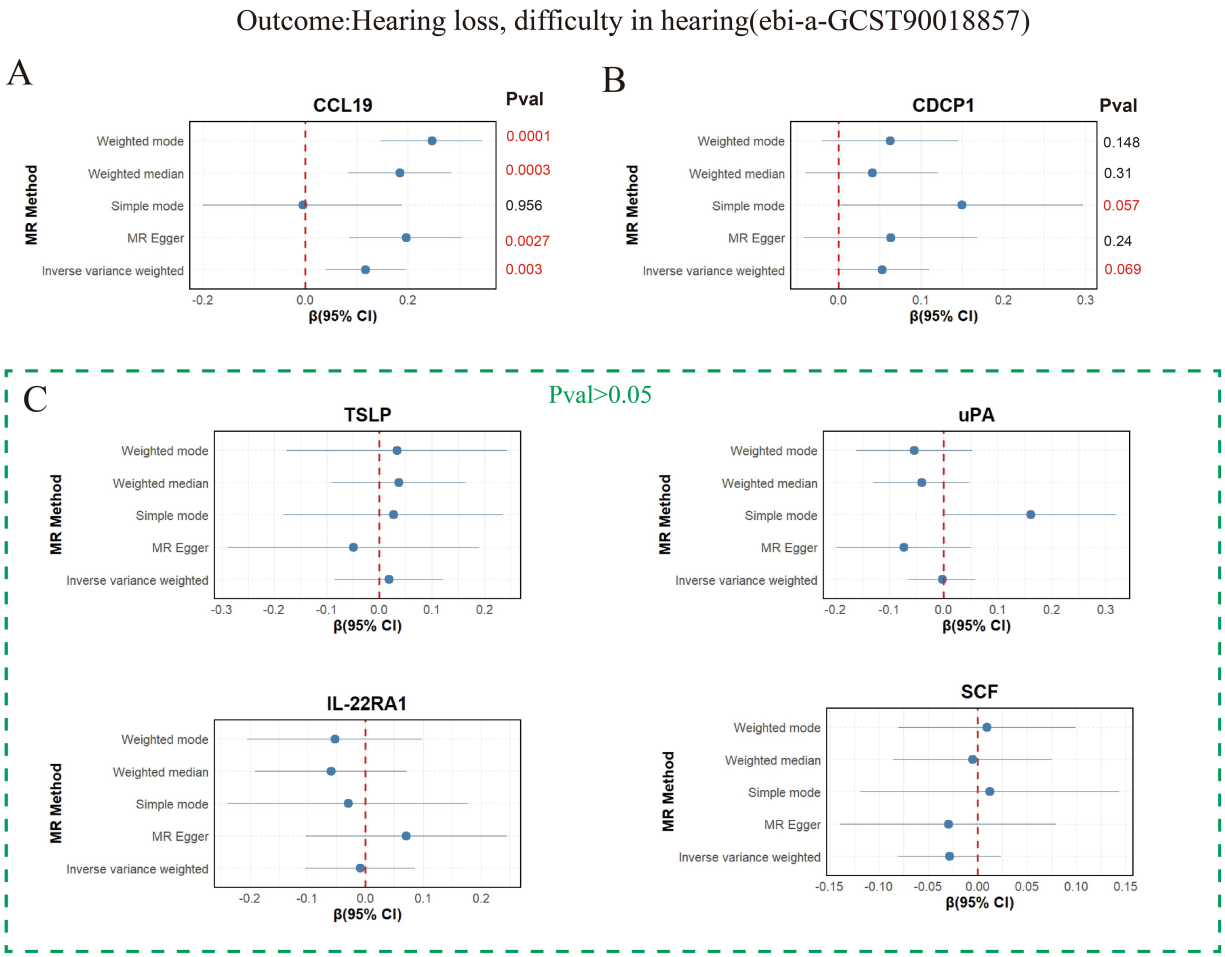
Mendelian analysis results of 6 inflammatory proteins and hearing loss, difficulty in hearing. **(A)** Mendelian analysis results of CCL19 and hearing loss, difficulty in hearing using five methods (weighted mode, weighted median, MR Egger, IVW, and simple mode). **(B)** Mendelian analysis results of CDCP119 and hearing loss, difficulty in hearing. **(C)** Mendelian analysis results of (uPA, TSLP, IL-22RA1, SCF) and hearing loss, difficulty in hearing.

Six inflammatory proteins (CCL19, CDCP1, uPA, TSLP, IL-22RA1, and SCF) associated with two or more types of hearing loss were further validated in the validation set “hearing loss, difficulty in hearing” (ebi-a-GCST90018857) (Supplementary Table S6A), and the results are presented in Figure 3. The weighted mode, weighted median, MR Egger, and IVW methods all supported the promoting effect of CCL19 on hearing loss (*p* < 0.05) (Figure 3A). It is worth noting that CDCP1 (*p*_IVW_ = 0.069) (Figure 3B) was not associated with hearing loss, and no associations were found between the remaining inflammatory proteins (uPA, TSLP, IL-22RA1, and SCF) and hearing loss in the validation set (Figure 3C). The *F*-statistics were greater than 10 (Supplementary Table S6B), and no horizontal pleiotropy was observed (Supplementary Table S6C).

### 3.2 Functional annotation, gene-based association and tissue analyses results

**Figure 4:**
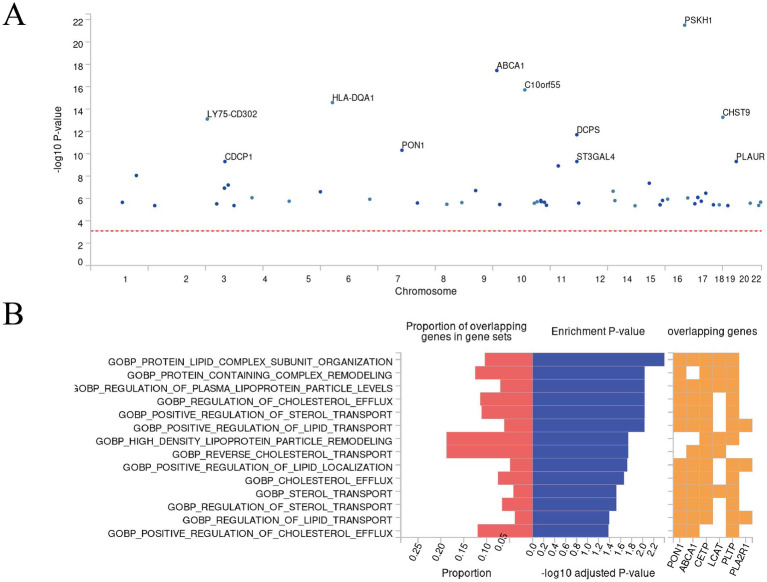
Results of FUMA’s “SNP2GENE” and “GENE2FUNC” analysis. **(A)** Manhattan plot. This Manhattan plot displays the gene-based test results computed by MAGMA. Input SNPs were mapped to 62 protein-coding genes, with the top 11 genes highlighted in the plot. The genome-wide significance threshold (red dashed line) was set at *p* = 0.05/62 = 8.065 ×10^−4^. **(B)** Biological processes enrichment plot. This functional enrichment plot, generated by FUMA’s GENE2FUNC analysis, illustrates the enrichment of GO biological processes (MsigDB) for a set of genes. The plot presents the proportion of overlapping genes in gene sets, enrichment *p*-values, and the specific overlapping genes. The left *x*-axis shows the proportion of overlapping genes in gene sets, while the right *x*-axis displays the −log_10_ adjusted *p*-value. The *y*-axis lists the GO biological processes. Red bars represent the proportion of overlapping genes in gene sets, and blue bars indicate the −log_10_ adjusted *p*-value. The orange grid highlights the overlapping genes for each process.

After gene annotation of SNPs related to SIHL-associated proteins, we identified 62 genes, including CDCP1 (Figure 4A). Detailed gene information is provided in Supplementary Table S7, and mapped gene-related information is available in Supplementary Table S8. Further “GENE2FUNC” enrichment analysis revealed that the biological processes are primarily associated with lipid metabolism, including protein-lipid complex subunit organization, protein complex remodeling, regulation of plasma lipoprotein particle levels, and positive regulation of lipid transport (Figure 4B). Lipid metabolism plays a crucial role in auditory function. A study showed that the loss of Bcl2 in the auditory cortex affects lipid metabolism, leading to decreased synaptic function and neurodegeneration (38). This finding aligns with our enrichment analysis results, further supporting the potential role of lipid metabolism in SIHL development.

GWAS catalog reported genes enrichment analysis showed that the identified genes (Supplementary Figure S7) are enriched in gene sets related to schizophrenia, serum lipid metabolism (including triglycerides), and C-reactive protein (Supplementary Table S9). Previous research has suggested that hearing impairment is a risk factor for mental health-related disorders, potentially due to factors such as loneliness and reduced theory of mind (39). Additionally, a cross-sectional study found that lipid and C-reactive protein levels are risk factors for hearing loss in older adults, with high dietary cholesterol intake increasing the risk of hearing loss (40).

Tissue enrichment analysis results showed that these genes are primarily expressed in the brain amygdala, cervical spinal cord, and spleen (Supplementary Figure S8). The amygdala, as a crucial region for emotional processing and auditory information integration, warrants further investigation in its association with SIHL. The enrichment in the spleen may reflect the potential role of the immune system in SIHL development. These findings provide new perspectives for understanding the molecular mechanisms of SIHL and point towards directions for future research and potential therapeutic strategy development.

### 3.3 Multi-omics network analysis results

**Figure 5:**
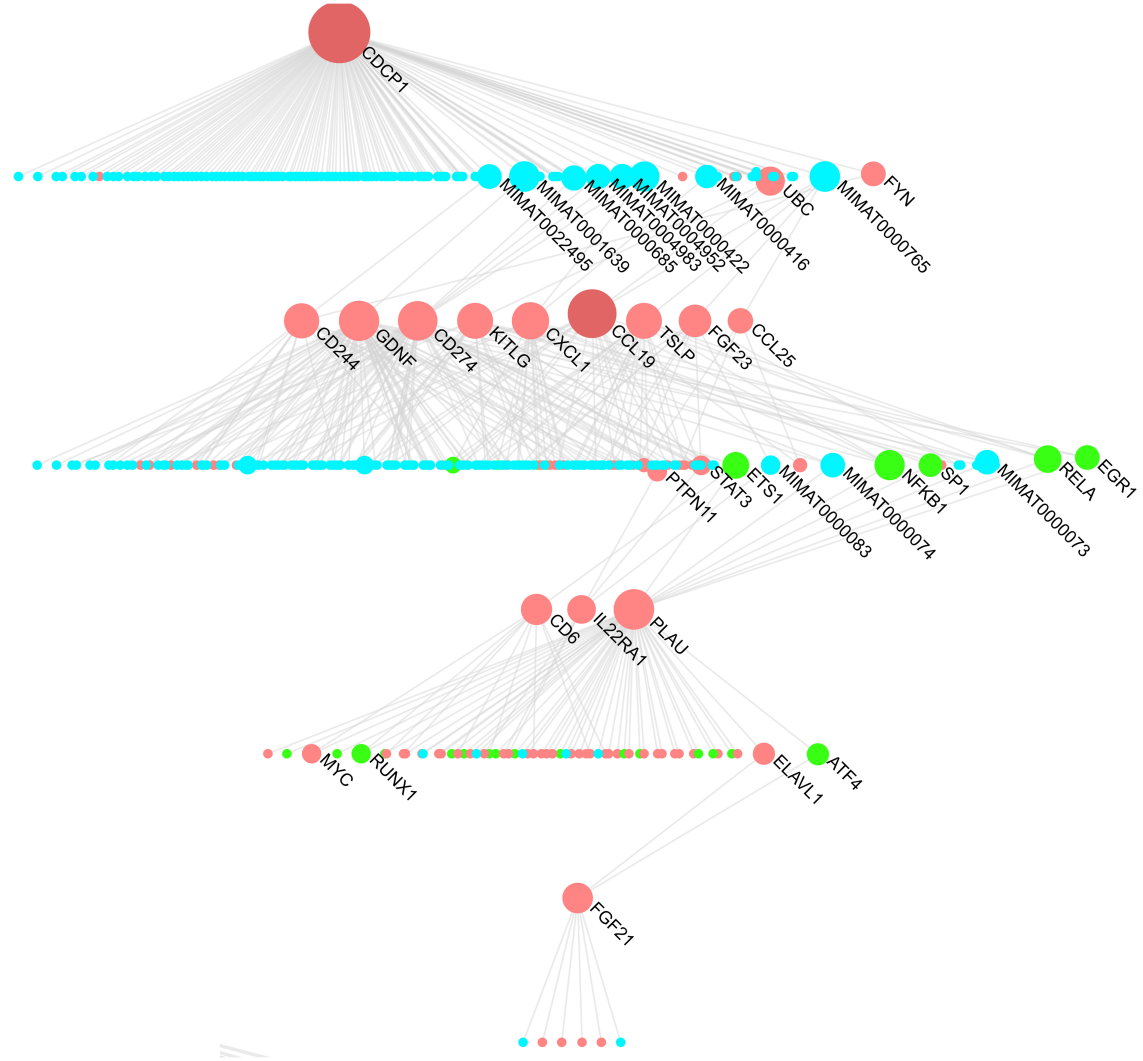
“Protein-miRNA-transcription factor” hierarchy for hearing-related proteins. Blue represents miRNAs, red represents proteins, and green represents transcription factors.

The multi-omics dendrogram (Figure 5) presents a “protein-miRNA-transcription factor” network after screening by MR analysis. CDCP1 protein, located at the top of the dendrogram, is a key node in this network and is associated with various downstream proteins. CCL19, TSLP, and other proteins occupy intermediary positions in the network.

The “protein-miRNA-transcription factor” network reveals the key regulatory miRNA factors (MIMAT0022495, MIMAT0001639, etc.) that link CDCP1 protein with downstream proteins (CCL19, TSLP, CCL25, FGF23, etc.). These shared miRNA regulatory factors may mediate common regulatory mechanisms that play crucial roles in diseases. For instance, MIMAT0000422 (hsa-miR-124-3p) and MIMAT0000416 (hsa-miR-1-3p) have been implicated in neurodegenerative diseases. Expression of hsa-miR-124-3p is downregulated in Alzheimer’s disease (41), while both hsa-miR-124-3p and hsa-miR-1-3p are downregulated in cerebellar neurodegeneration (42). These neurodegenerative changes may lead to hearing loss (43, 44).

In addition to identifying key miRNAs, the “protein-miRNA-transcription factor” network also identified critical proteins and transcription factors such as ubiquitin-conjugating enzyme E2 C (UBC), early growth response 1 (EGR1), signal transducer and activator of transcription 3 (STAT3), nuclear factor kappa-B (NF-*κ*B), and the MYC gene. Studies have shown that the STAT3 pathway is associated with various ototoxic hearing losses, while the NF-*κ*B pathway promotes ARHL. Caffeine can improve this process by downregulating the NF-*κ*B inflammatory pathway (45).

### 3.4 Drug repurposing prediction results

**Figure 6:**
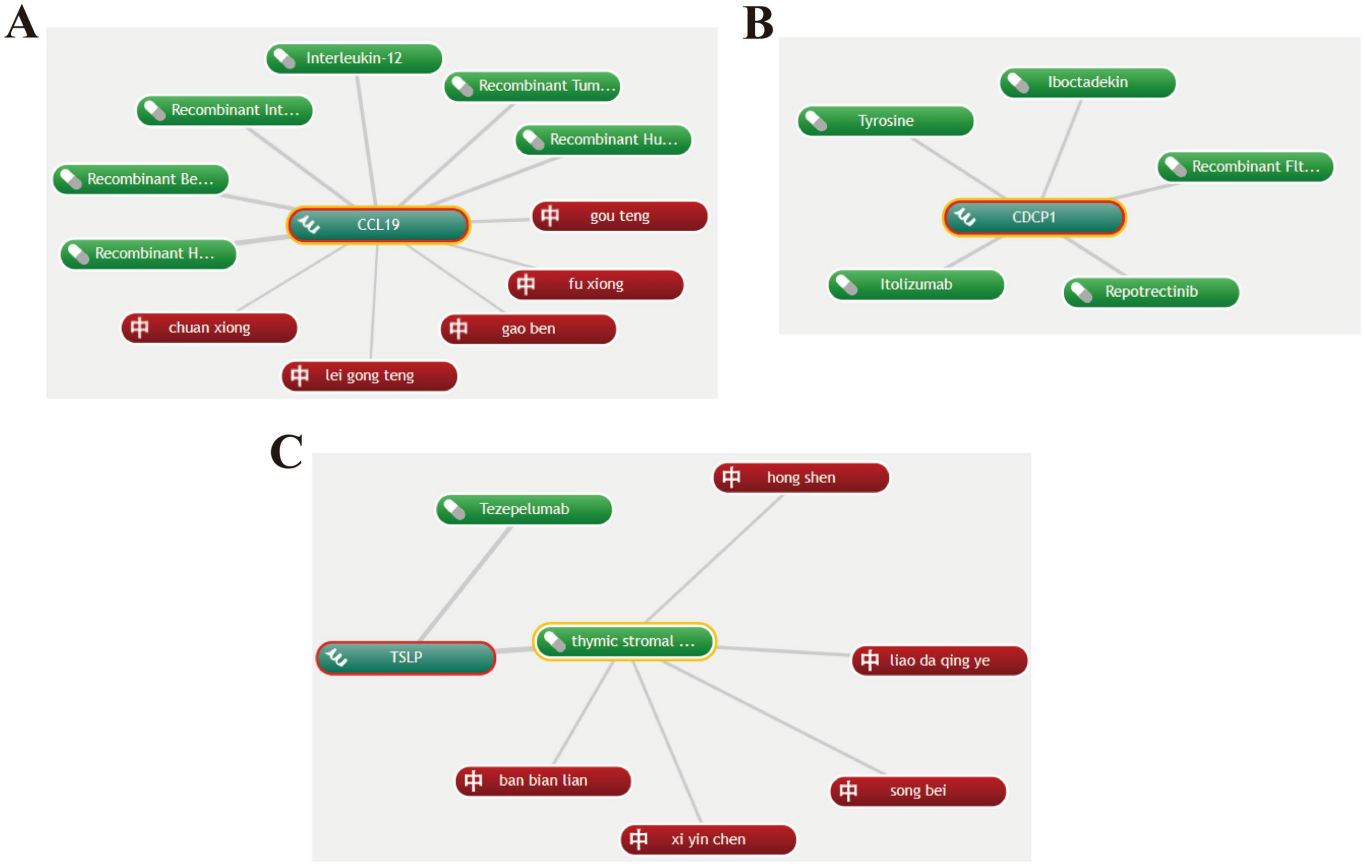
Potential drugs targeting CCL19, CDCP1 and TSLP. **(A)** Potential drugs targeting CCL19. **(B)** Potential drugs targeting CDCP1. **(C)** Potential drugs targeting TSLP. Green represents Western medicines, and red represents Chinese herbal medicines.

To identify potential drug repurposing opportunities, we performed drug target analysis on the core proteins CCL19, CDCP1, and TSLP. The “CoreMine” database revealed six drugs targeting CCL19, including recombinant human 6Ckine, recombinant beta chemokine, recombinant interleukin-7, interleukin-12, recombinant tumor necrosis factor-beta, and recombinant human macrophage inflammatory protein-1 beta. Two of these drugs were found to be in clinical trials according to the DrugBank database. Interleukin-7 has been used in trials investigating the treatment of metastatic breast cancer (46). Additionally, cetuximab and recombinant interleukin-12 are being used in a trial treating patients with recurrent, metastatic, or unresectable squamous cell carcinoma of the head and neck (detailed information available in DrugBank). Traditional Chinese medicines targeting CCL19 include Chuanxiong Rhizoma, Uncariae Ramulus Cumuncis, etc. (Figure 6A).

Based on the drug repurposing results from “CoreMine,” the drugs targeting CDCP1 include tyrosine, iboctadekin, itolizumab, repotrectinib, and recombinant Flt3 ligand. Among these, tyrosine can act as a mood elevator and antidepressant, while also improving memory and increasing mental alertness (47, 48) (Figure 6B).

Tezepelumab, a human monoclonal IgG2λ antibody that blocks TSLP, is the primary drug targeting TSLP and is mainly used for the treatment of asthma (49). Traditional Chinese medicines targeting TSLP include *Carthami flos* and *Lobeliae chinensis* Herba (Figure 6C).

### 3.5 Molecular docking results

**Figure 7:**
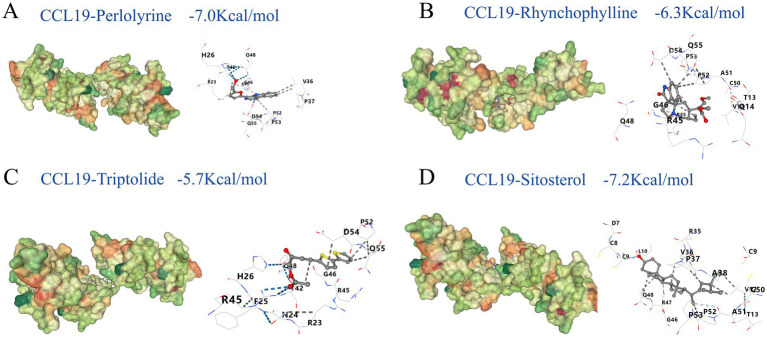
Molecular docking results. This figure shows the molecular docking results of perlolyrine **(A)**, rhynchophylline **(B)**, triptolide **(C)** and sitosterol **(D)** with CCL19. The binding energies are −7.0, −6.3, −5.7, −7.2 kcal/mol for **A–D**, respectively. In each subfigure, the 3D structure displays the interaction between the ligand and the protein, with key amino acid residues and interaction types labeled. The blue dashed lines represent hydrogen bonds; white dashed lines represent weak hydrogen bonds; black dashed lines represent hydrophobic contacts.

After identifying traditional Chinese medicines targeting CCL19 through “CoreMine,” including Chuanxiong Rhizoma, Uncariae Ramulus Cumuncis, Ligustici Rhizoma Et Radix, and Tripterygii Radix, molecular docking analysis was performed to assess the binding affinity of their active components with CCL19 (Supplementary Table S10). The results revealed promising interactions for several compounds: perlolyrine (−7.0 kcal/mol), rhynchophylline (−6.5 kcal/mol), triptolide (−5.7 kcal/mol), and sitosterol (−7.2 kcal/mol), as illustrated in Figure 7. These binding energies indicate stable interactions between the compounds and CCL19, with lower values suggesting stronger potential drug effects at the target site. Notably, binding energies below −7.0 kcal/mol are generally considered to indicate strong binding affinity and potential biological activity (50). Moreover, the drugs we identified exhibit promising binding affinity with CDCP1 and TSLP (Supplementary Table S11). These findings provide insights into the potential efficacy of these TCM components in modulating inflammatory proteins-related pathways involved in hearing loss, laying a foundation for further investigation into their therapeutic potential and mechanisms of action in addressing hearing impairments.

## 4 Discussion

Our MR analysis revealed CCL19 as a significant risk factor for both SNHL and OHL (Figure 2). This association was further validated in datasets focusing on hearing loss and difficulty in hearing (Figure 3A), demonstrating robust reliability. CCL19 is a chemokine that serves as a crucial mediator in immune responses and inflammatory processes (51). The immune homeostasis of the inner ear is predominantly maintained by resident macrophages distributed across various cochlear structures, including the spiral ligament, spiral ganglion, and stria vascularis. These macrophages serve multiple functions: they detect and eliminate tissue debris and pathogens through phagocytosis, while simultaneously orchestrating inflammatory responses and tissue repair by secreting inflammatory cytokines and chemokines (52). The interaction between CCL19 and CCR7 receptors on macrophage surfaces triggers downstream signaling cascades, thereby modulating macrophage chemotaxis, migration, and functional polarization (53). Accumulating evidence strongly supports CCL19’s role as a risk factor in hearing loss development. Experimental studies have demonstrated that noise exposure significantly elevates CCL19 expression in GRAIL wild-type mice, whereas GRAIL gene deletion reduces CCL19 levels and subsequently attenuates hearing loss, suggesting CCL19 as a promising therapeutic target for noise-induced hearing loss (54). Further supporting evidence comes from gene expression analyses of rat endolymphatic sac (ES), which revealed that dysregulation of CCL19-related genes may contribute to autoimmune inner ear disorders (55). Moreover, CCL19 has been implicated in inner ear aging processes. Through comprehensive comparative analyses of age-related differential gene expression and aging signatures in mouse cochlea, researchers identified CCL19 as a high-risk gene associated with age-related hearing decline (56).

Notably, CCL2 plays a pivotal role in the pathogenesis of hearing loss. Following cochlear injury, such as noise exposure, CCL2 levels are significantly elevated in the organ of Corti and surrounding inner ear tissues (57). Further investigations have demonstrated that anti-inflammatory medications effectively reduce CCL2 levels and ameliorate hearing loss (58, 59). The involvement of inflammatory and immune responses in cochlear damage is evidenced by the upregulation of inflammation/immune-related genes and increased immune cell infiltration following various types of injury (60). The mitochondrial toxin 3-nitropropionic acid (3-NP) induces acute mitochondrial dysfunction and activates the IL-6/CCL2 inflammatory pathway, leading to secondary inflammatory responses in the cochlear lateral wall. CCL2 has emerged as a potential therapeutic target for treating injuries resulting from acute mitochondrial dysfunction in the cochlear lateral wall (61). Additionally, studies have observed upregulation of CCL2-related genes in aged mouse cochlea, suggesting its association with age-related hearing loss (62).

Our MR analysis identified CDCP1 as a risk factor and TSLP as a protective factor for both SIHL and OHL (Figure 2). Although previous research found no significant association between CDCP1 and persistent tinnitus (63), the impact of CDCP1 on hearing remains largely unexplored. Our multi-omics analysis (Figure 5) reveals that CDCP1 regulates the downstream transcription factor STAT3 (64), which differentially modulates inflammatory and apoptotic signaling in the cochlea and regulates TRPV1 channels (65). TRPV1 has been established as a mediator for aminoglycoside entry into hair cells and is implicated in cisplatin-induced ototoxicity (66, 67). Suppression of oxidative stress or inflammation reduces TRPV1 channel expression, thereby preventing cochlear damage and hearing loss (65). Furthermore, studies have demonstrated that apelin-13 and capsaicin can protect against cisplatin-induced ototoxicity through STAT3 regulation (68, 69), suggesting the need for further investigation into the relationship between STAT3 and hearing loss, particularly in the context of ototoxicity. Research on TSLP’s role in hearing loss is limited; however, studies have shown significantly elevated TSLP levels in children with chronic serous otitis media, where it initiates and maintains local inflammatory responses in the Eustachian tube and may contribute to middle ear effusion in non-atopic patients (70).

Our functional enrichment analysis revealed that SIHL-associated proteins were significantly enriched in lipid metabolism pathways (including serum triglycerides) (Figure 4B) and schizophrenia-related pathways (Supplementary Table S9). The multi-omics network analysis (Figure 5) further demonstrated the role of inflammation-related miRNAs in neurodegenerative diseases. High-fat diets can induce systemic inflammation (71), which compromises blood-brain barrier (BBB) permeability and facilitates inflammatory factor infiltration into the brain, potentially leading to neurodegeneration (72, 73). Our tissue analysis identified enrichment of these inflammatory factors in the amygdala (Supplementary Figure S8), a region crucial for anxiety, post-traumatic stress disorder (PTSD), various psychological disorders, and auditory processing. The release of inflammatory factors can impair emotional regulation in the amygdala, increasing the risk of anxiety and depression, thereby exacerbating hearing loss (74). Chronic inflammation may also disrupt the amygdala’s stress response system, elevating the risk of neurodegenerative diseases such as Alzheimer’s disease (75, 76). Additionally, serum triglycerides can affect cochlear blood supply (77), compromise blood-labyrinth barrier integrity, leading to reduced oxygen supply to the inner ear and subsequent ischemic damage to hair cells (78). Our study highlights the connections between inflammation and various types of hearing loss, exploring the crosstalk between inflammation, downstream signaling pathways, and miRNAs, as well as their localization in the brain, thereby advancing our understanding of inflammation-mediated hearing loss mechanisms. Furthermore, we identified potential small-molecule drugs targeting these proteins (Figure 7), potentially offering new therapeutic strategies for hearing loss treatment.

The superiority of this study lies in its comprehensive investigation of the relationship between inflammation and hearing loss. Unlike previous studies that focused on a limited number of inflammatory markers (such as CRP and IL-6) (13, 79), this study expanded the scope to 91 inflammatory proteins. The study benefits from a comprehensive proteomic profile, a diverse set of outcomes (SNHL, SIHL, and OHL), minimal confounding and bias, and validation using a large cohort dataset, resulting in more robust findings and a more complete network of inflammation and hearing loss. Furthermore, Functional annotation, tissue analyses, and multi-omics network analysis provide further insights into the disease mechanisms. Drug repurposing and molecular docking analysis also offers potential directions for clinical treatment.

However, this study has some limitations. The population in this study primarily consists of Europeans, and further generalization should be made with caution. Due to the limitations of the available data, we were unable to further subdivide SNHL into age-related hearing loss and noise-induced hearing loss (80), which we believe would greatly benefit personalized treatment and should be a direction for future research. Although this study emphasizes the key role of CCL19 in hearing loss, current research findings suggest that further investigation of the CCL family, especially CCL2, is necessary. While Mendelian randomization can largely avoid the influence of confounding factors on the outcomes, further validation through animal experiments and clinical trials is still crucial.

## 5 Conclusion

This study investigated the causal relationships between various inflammatory proteins (including CCL19, CDCP1, and TSLP) and multiple types of hearing loss (SNHL, SIHL, and OHL). The research focused on downstream inflammatory signaling pathways and miRNA crosstalk, integrating enrichment and tissue analyses to construct an “inflammatory protein-hearing loss” network. Mechanisms by which inflammatory proteins lead to hearing loss were explored, and small molecule drugs targeting these proteins were identified. Future research directions encompass more refined analyses for different types of hearing loss, in-depth exploration of the relationship between CCL family members (particularly CCL2) and hearing loss, and prospective studies in both animal models and human populations. These efforts will enhance our understanding of the pathogenesis of hearing loss and provide additional leads for drug development.

## Data Availability

The datasets presented in this study can be found in online repositories. The FinnGen database data are available at https://www.finngen.fi/en/access_results and the GWAS summary datasets are available at https://gwas.mrcieu.ac.uk/.

## Footnotes

^1^ https://www.finngen.fi/en/access_results

^2^ https://www.omicsnet.ca/

^3^ https://www.ncbi.nlm.nih.gov/

^4^ https://www.coremine.com/

^5^ https://go.drugbank.com/

^6^ https://pubchem.ncbi.nlm.nih.gov/

^7^ https://www.uniprot.org/

^8^ http://cadd.labshare.cn/cb-dock2/
