# Supplementary material for: Investigating the causal relationship between inflammation and multiple types of hearing loss: a multi-omics approach combining Mendelian randomization and molecular docking
Source: Front Neurol. 2024 Nov 28;15:1422241. doi: 10.3389/fneur.2024.1422241 (PMC11638537; doi:10.3389/fneur.2024.1422241)
Supplement: Supplementary file 1 [file Table_1.DOCX]

# Supplementary Figures


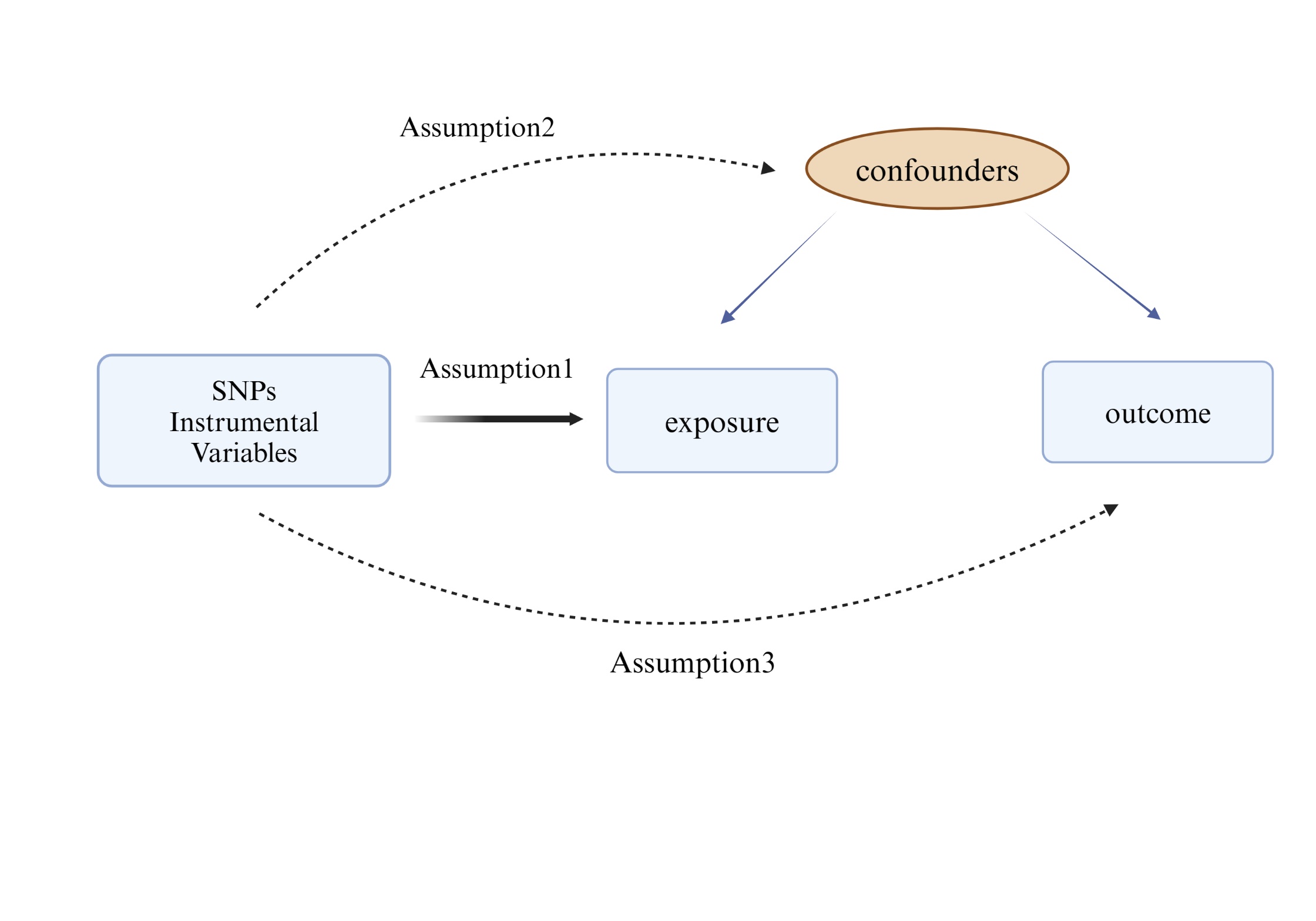


**Supplementary Figure 1.** The core assumptions of Mendelian Randomization.

Assumption 1: The genetic variants must exhibit a robust association with the intended exposure.

Assumption 2: The instrumental genetic variants should operate independently of any confounders that could affect the exposure-outcome relationship.

Assumption 3: Genetic variants should exert influence on the outcome exclusively through their effect on the designated exposure.


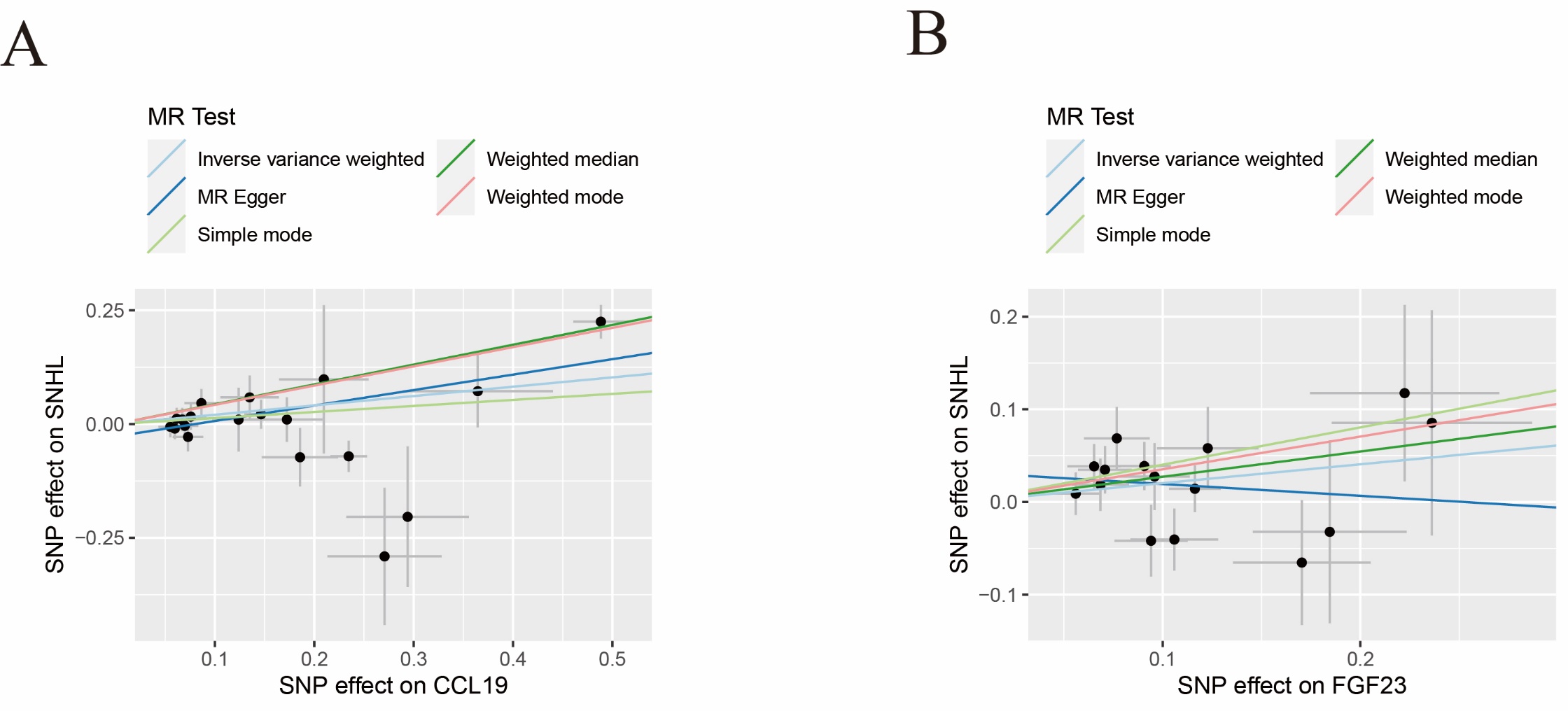


**Supplementary Figure 2.** Scatter plots of the causal effect results of inflammation proteins on SNHL. (A) CCL19, (B) FGF23.


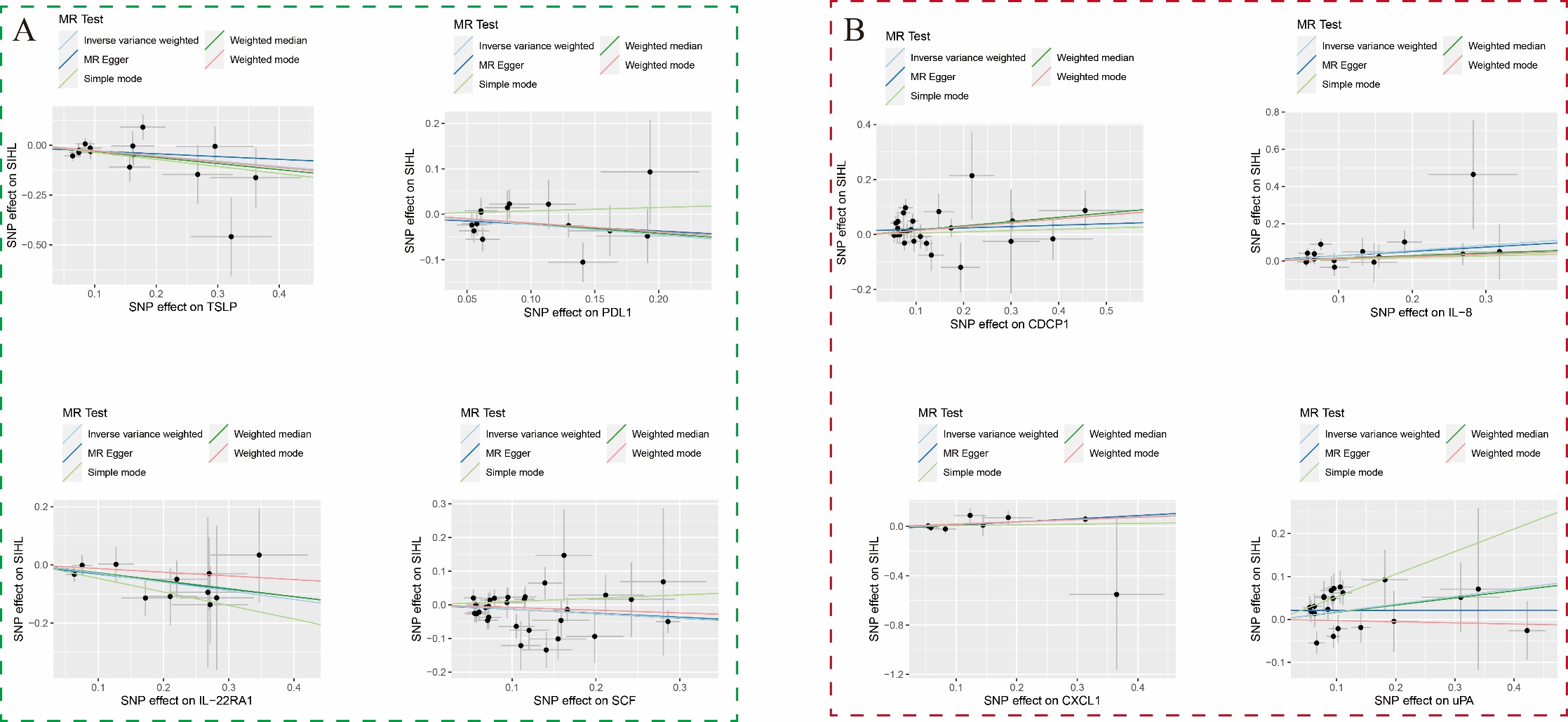


**Supplementary Figure 3.** Scatter plots of the causal effect results of inflammation proteins on SIHL. (A) Four protective proteins associated with SIHL: TSLP, PDL1, IL-22RA1, SCF, (B) Four risk proteins associated with SIHL: CDCP1, IL-8, CXCL1, uPA.

**
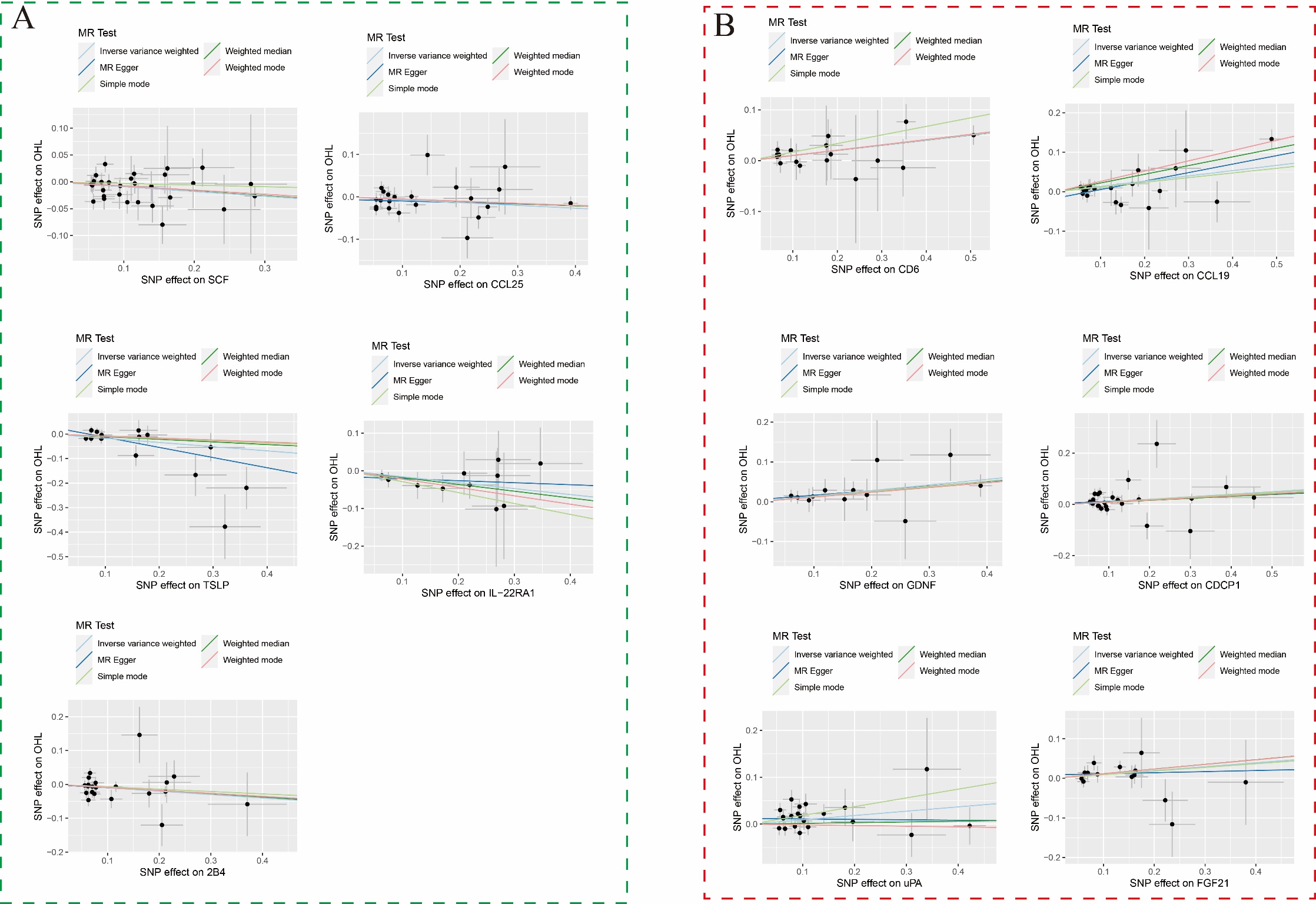
**

**Supplementary Figure 4.** Scatter plots of the causal effect results of inflammation proteins on OHL. (A) Five protective proteins associated with OHL: SCF, CCL25, TSLP, IL-22RA1, 2B4, (B) Six risk proteins associated with SIHL: CD6, CCL19, GDNF, CDCP1, uPA, FGF21.

**
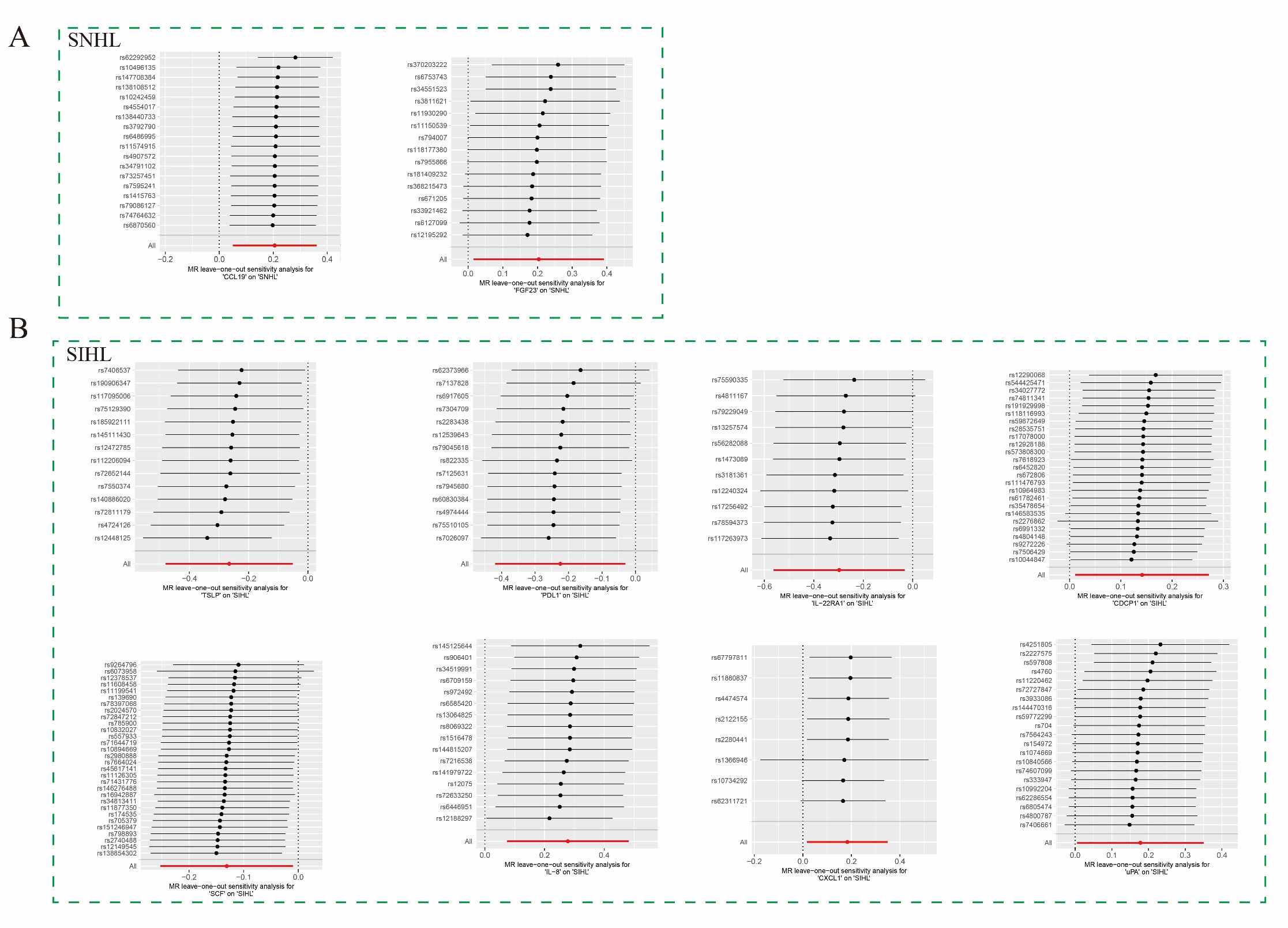
**

**Supplementary Figure 5.** The leave-one-out sensitivity analysis plots for inflammatory proteins on SNHL and SIHL. (A)The leave-one-out analysis plots for inflammatory proteins on SNHL. (B) The leave-one-out analysis plots for inflammatory proteins on SIHL.


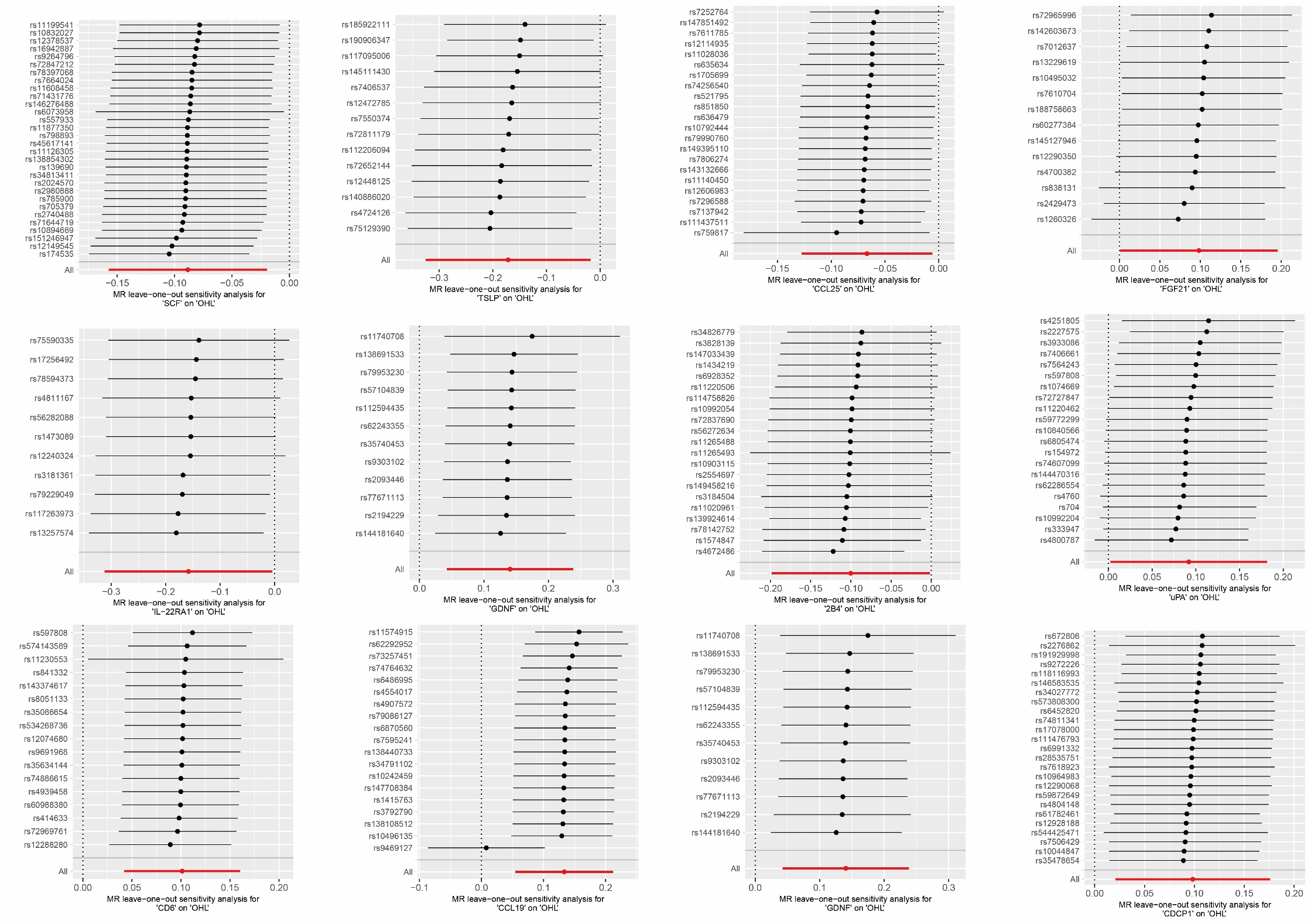


**Supplementary Figure 6.** The leave-one-out sensitivity analysis plots for inflammatory proteins on OHL.


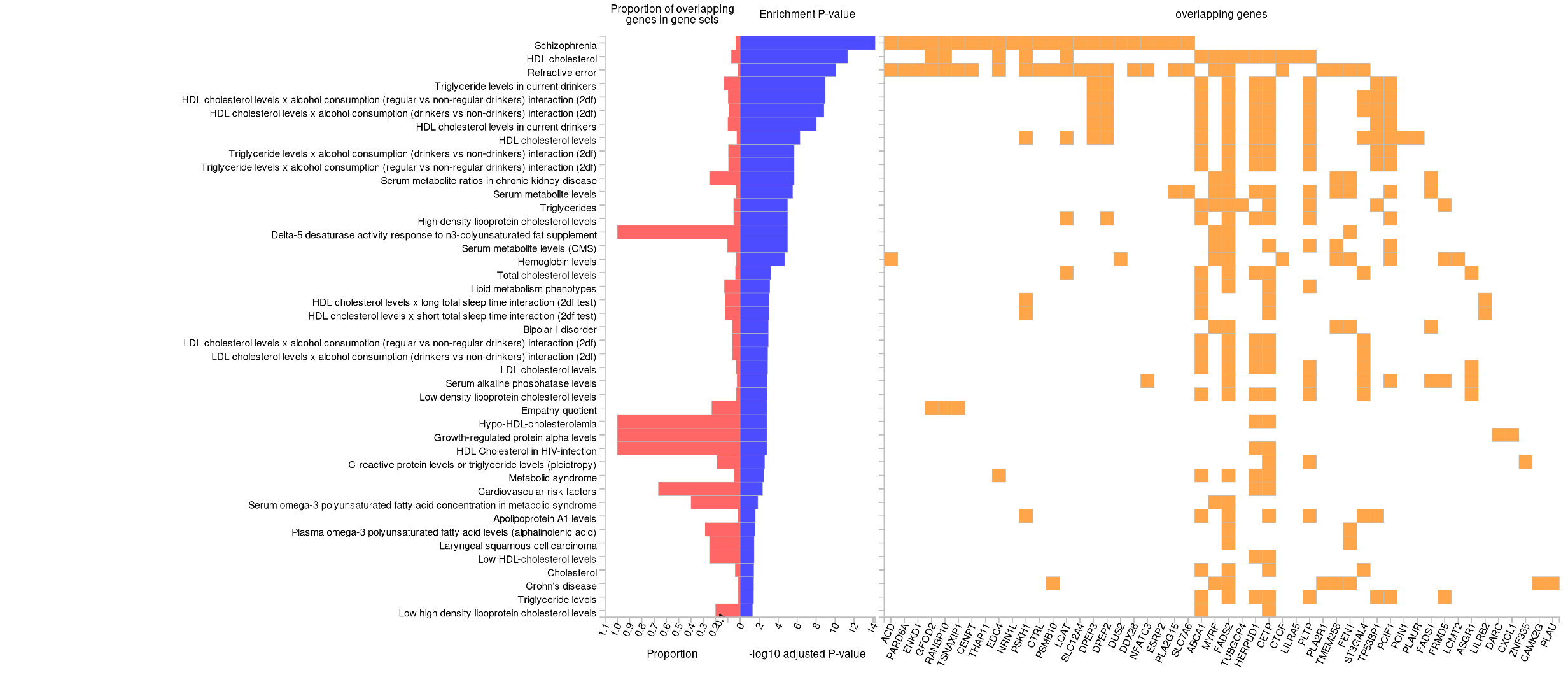


**Supplementary Figure 7.** GWAS Catalog Reported Genes Enrichment in Gene Sets Related to SIHL-Associated Proteins.

This figure displays the results of a gene set enrichment analysis, where genes associated with various traits (e.g., lipid levels, alcohol consumption) from the GWAS Catalog are tested for enrichment in gene sets linked to proteins related to SIHL.

**
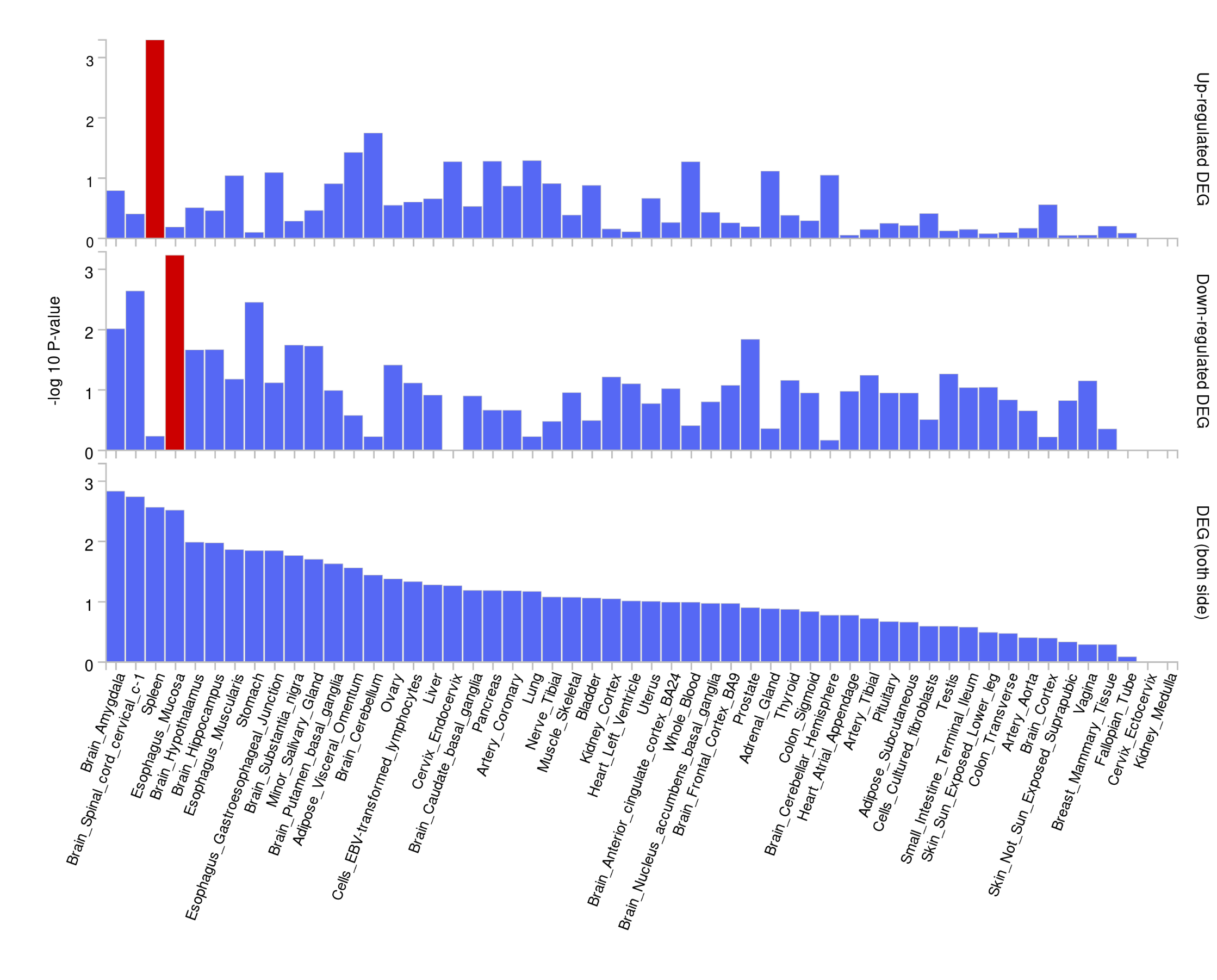
**

**Supplementary Figure 8.** Tissue-Specific Enrichment of Differentially Expressed Genes.
